# Impact of limb ischemic preconditioning on the incidence of vein thrombosis in patients with peripherally inserted central catheter

**DOI:** 10.3389/pore.2024.1611596

**Published:** 2024-11-14

**Authors:** Han Zhao, Changhua Kou, Hao Zhao, Qing Liu, Maosheng He, Cong Wang, Saisai Zhu, Li Ma, Yun Wang

**Affiliations:** ^1^ Clinic for Kidney and Hypertensive Diseases, Hanover Medical School, Hanover, Germany; ^2^ Department of Hepatobiliary Pancreatic Center, Xuzhou City Central Hospital, The Xuzhou School of Clinical Medicine of Nanjing Medical University, The Affiliated Hospital of the Southeast University Medical School, Xuzhou, Jiangsu, China; ^3^ Department of Vascular Surgery, Xuzhou City Central Hospital, The Affiliated Hospital of the Southeast University Medical School, Xuzhou, Jiangsu, China; ^4^ Department of Gynecology Department, Xuzhou City Central Hospital, The Xuzhou School of Clinical Medicine of Nanjing Medical University, The Affiliated Hospital of the Southeast University Medical School, Xuzhou, Jiangsu, China; ^5^ Department of Color Ultrasound Department, Xuzhou City Central Hospital, The Xuzhou School of Clinical Medicine of Nanjing Medical University, The Affiliated Hospital of the Southeast University Medical School, Xuzhou, Jiangsu, China; ^6^ Department of Thyroid and Breast Surgery, Xuzhou City Central Hospital, The Affiliated Hospital of the Southeast University Medical School, Xuzhou, Jiangsu, China

**Keywords:** tumor, limb ischemic preconditioning, peripherally inserted central catheter, vein thrombosis, PICC-related complications

## Abstract

**Background:**

Peripherally inserted central catheters (PICC) are increasingly used in clinical practice, which also leads to an increased incidence of PICC-related thrombosis. Local thrombus formation could be prevented by limb ischemic preconditioning (IPC). This study aimed to determine whether IPC can prevent deep vein thrombosis in patients with PICC.

**Methods:**

A total of 600 breast cancer patients receiving PICC were randomized into two groups between July 2016 and July 2018 at the Department of Radiation Oncology. In the preconditioning group, 5 min of ischemic preconditioning was performed three times before PICC, whereas no preconditioning was performed in the sham group. The coagulation function levels, the PICC-related complications, the length of hospital stay, the cost of hospitalization, and the satisfaction of patients were compared.

**Results:**

The coagulation function levels of patients in the preconditioning group were more normal than in patients from the sham group. In total, 56/300 patients in the sham group had presence of PICC-related thrombosis, with only 23/300 in the IPC group, with no significant difference in other complications between the two groups. However, a longer hospital stay was observed in the sham group compared to the IPC group. Moreover, the cost of hospitalization was also reduced in the IPC group, which also improved the satisfaction of patients.

**Conclusion:**

Limb ischemic preconditioning may attenuate the severity of vein thrombosis in patients with PICC, which contributes to reducing the incidence of PICC-related thrombosis in clinical practice.

## Introduction

Peripherally inserted central catheters (PICCs) are a form of intravenous access in which the catheter is directed from a peripheral vein to a large heart vein. These catheters can be used for an extended period of time and minimize the stimulation of drugs to blood vessels [1–3]. PICC is now increasingly used in clinical practice owing to its safety and cost-effectiveness. However, PICC also involves some complications, such as puncture failure, phlebitis, infection, and thrombosis. PICC-related thrombosis is a severe complication that may lower the quality of life of patients, increase hospitalization costs, and even increase mortality [4, 5]. Patients and doctors are both concerned with PICC-related thrombosis. To reduce the incidence of PICC-related thrombosis, a number of studies have investigated the risk factors and prevention measures for thrombosis in patients with PICC.

Remote ischemic preconditioning (RIPC) is a process by which brief periods of ischemia trigger protective effects on remote organs or tissues. RIPC is widely used in patients to protect organs or tissues prone to ischemic injury, including the liver, brain, myocardium, and so on [6–8]. Research revealed that limb ischemic preconditioning (IPC) could promote thrombus recanalization and reduce the incidence of thrombus in rats [9]. The three main factors necessary for thrombosis formation include stasis of blood, vessel wall injury, and platelet aggregation [10, 11]. Multiple studies have demonstrated that remote IPC before radiofrequency catheter ablation significantly reduced the platelet activation and reactivity of patients with paroxysmal atrial fibrillation [12].

Based on the above content, 600 breast cancer patients receiving PICC in our department were randomized into two groups (sham group and preconditioning group) with 1:1 allocation. Then, the efficacy of catheterization, the coagulation function levels, the incidence of thrombosis, the length of hospital stay, the cost of hospitalization, and the satisfaction of patients were compared between the two groups. The results may verify our hypothesis that limb ischemic preconditioning could play a protective role in the incidence of thrombus after PICC, which could contribute to reducing the incidence of PICC-related thrombosis in clinical practice.

## Methods

### Experiment design

This single-center, parallel, and double-blind trial included 600 breast cancer patients receiving PICC, who were randomly assigned to two groups with 1:1 allocation. The patients in the preconditioning group were subjected to 5 minutes of ischemic preconditioning three times before PICC, whereas no preconditioning was performed in the sham group. The study was conducted in accordance with the Declaration of Helsinki. The studies involving human participants were reviewed and approved by Xuzhou Central Hospital Biomedical Research Ethics Review Committee (Permit Number: XZXY-LJ-20161210-032). The patients/participants provided their written informed consent to participate in this study.

### Inclusion and exclusion criteria

The inclusion criteria were as follows: 1) Adults older than 18 years and younger than 70 years; 2) Patients all underwent mastectomy for breast cancer and needed chemotherapy; 3) Patients were diagnosed with breast cancer with TNM stages II-III; 4) The patients volunteered for PICC, signed the treatment consent form, and volunteered to participate in this project; 5) Normal coagulation function, and 6) No other past Medical History. The exclusion criteria were as follows: 1) Patients older than 70 years or younger than 18 years; 2) Patients who underwent mastectomy for breast cancer but did not require chemotherapy; 3) Patients were diagnosed with breast cancer with TNM stages other than II-III; 4) Patients refused to undergo PICC or refused to enter the project; 5) Abnormal coagulation function, and 6) The presence of other past Medical History.

### Surgical procedures

We used the PICC Center venous catheter (BARD, United States), with specifications: Groshong NXT ClearVue 4Fr single-lumen basic kit, including a micro-introducer sheath. The operating procedure for PICC was carried out as described by Amerasekera SS et al. PICC insertion was carried out by the same vascular access team. The success of PICC placement was confirmed based on the color Doppler ultrasound and interventional radiology [4].

### Treatment of patients in the sham and IPC group

Both patients in the sham and IPC groups had blood pressure cuffs around their arm while undergoing PICC. The cuff was inflated to 200 mm Hg three times for 5 min in the IPC group and was released to allow reperfusion between the cycles. No preconditioning was performed in patients of the sham group.

### Measurement of coagulation function levels

Blood samples of patients were used for coagulation function tests, which were collected from the vena mediana cubiti before PICC and at days 14, 30, 60, and 90 after PICC. The coagulation function test included prothrombin time (PT), thrombin time (TT), activated partial thromboplastin time (APTT), fibrin(-ogen) degradation products (FDP), and D-Dimer. All tests were performed in the clinical laboratory of Xuzhou City Central Hospital.

### The observation of PICC-related complications

Local inflammation, bloodstream infection, occlusion, dislocation, PICC-related thrombosis, local bleeding, pulmonary artery embolism, and local allergic reaction were observed and recorded. Patients were subjected to color Doppler ultrasound (SIEMENS, ACUSON S3000) to assess the incidence of thrombosis at days 14, 30, 60, and 90 after PICC.

### Statistical analysis

All data are expressed as the mean ± standard deviation. The data were analyzed using variance, q-test, and Student’s t-tests. *P* < 0.05 was considered statistically significant. SPSS14.0 software was used for all statistical analyses.

## Results

### Evaluation of PICC

**FIGURE 1:**
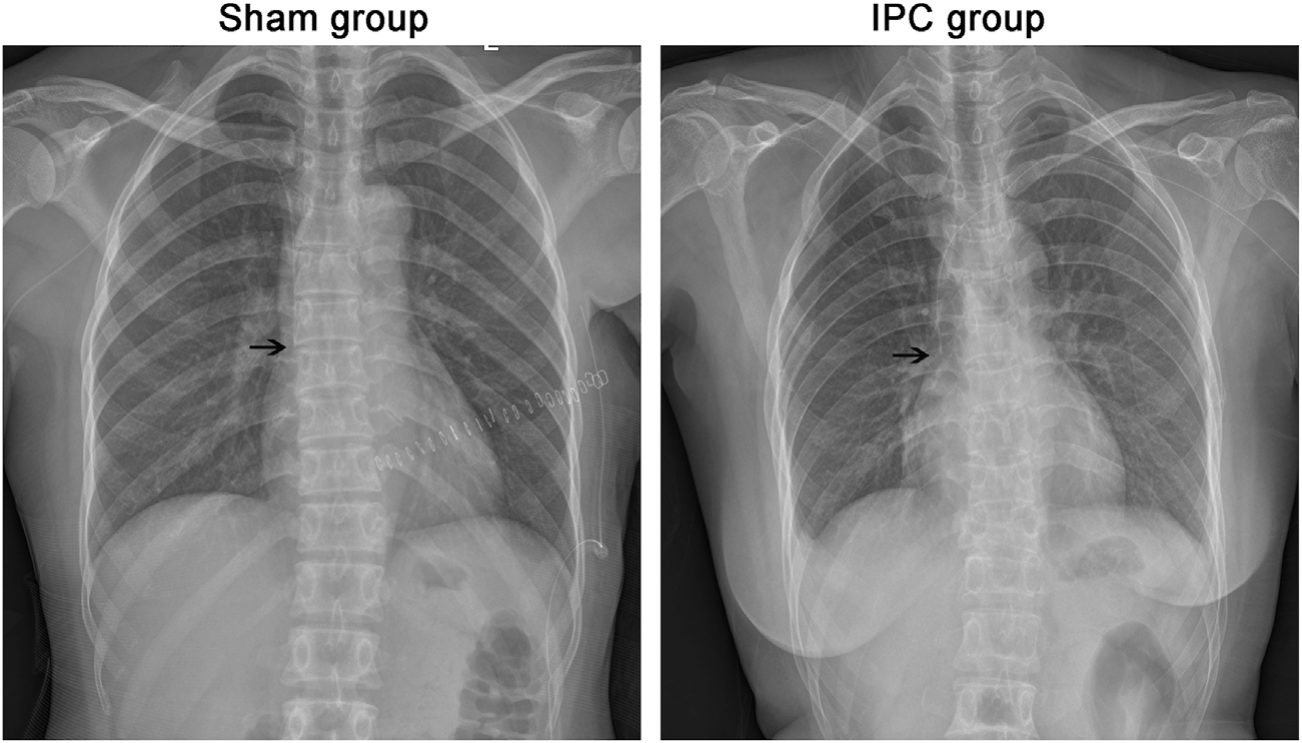
Evaluation of PICC. After the operation, all the cases were taken x-ray to determine the position of the catheter tip. The end of PICC catheter was in normal position between T5 and T7. The black arrow shows the end of the catheter.

After the catheter insertion, an X-ray was taken to determine the position of the catheter tip. Patient characteristics, procedural details, and the results of X-rays are shown in Figure 1 and Table 1. All patients had no statistically significant differences in baseline information, except for intraoperative blood loss. However, since all patients underwent PICC catheter placement 1 month after surgery, the difference in intraoperative blood loss did not affect their coagulation function and thrombus formation.

**TABLE 1:** Patient characteristics, procedural details, and the results of X-rays.

		IPC	Sham	T value	*P*-value
General data	Age (years)	53.36 ± 15.61	54.31 ± 15.06	0.761	0.447
	BMI	22.78 ± 3.44	22.96 ± 3.58	0.629	0.53
				X^2^ value	*P*-value
	Right/left breast, n (%)	147/153, (49/51)	135/165, (45/55)	0.96	0.33
Clinical data	Patients undergoing first resection, n (%)	300, (100)	298, (99.3)	2.01	0.16
	TNM II stage, n (%)	211, (70.3)	202, (67.3)	0.63	0.43
	TNM III stage, n (%)	89, (29.7)	98, (32.7)	0.63	0.43
				T value	*P*-value
Surgical data	Surgery duration (min)	104.82 ± 38.27	102.44 ± 38.81	0.755	0.45
	Intraoperative blood loss (mL)	43.97 ± 27.79	50.7 ± 27.95	2.96	<0.05*
				X^2^ value	*P*-value
	Halsted surgery, n (%)	17, (5.7)	18, (6)	0.03	0.86
	Auchincloss surgery, n (%)	155, (51.7)	146, (48.7)	0.54	0.46
	Patey surgery, n (%)	117, (39)	124, (41.3)	0.34	0.6
	Conserving surgery, n (%)	11, (3.7)	12, (4)	0.05	0.83
Evaluation of PICC	One-time success, n (%)	300, (100)	300, (100)	1	1

*p < 0.05 compared with the sham group.

### Serum coagulation function levels

As shown in Table 2, blood samples were collected to evaluate the values of FDP and D-Dimer, and the coagulation function by PT, APTT, TT, and FIB. As shown in Table 2, all of the values were within the normal reference range, but the values of FDP (days 14, 30, 60, and 90) and D-Dimer (day 60) in the sham group were significantly higher than those in the IPC group (FDP: mg/L, sham group, d14: 3.07 ± 1.91, d30: 3.11 ± 1.74, d60: 2.31 ± 1.51, d90: 2.20 ± 1.62, IPC group, d14: 2.53 ± 1.57, d30: 2.54 ± 1.69, d60: 2.01 ± 1.76, d90: 1.96 ± 1.58. D-Dimer: mg/L FEU, sham group, d60: 0.32 ± 0.26, IPC group, d60: 0.26 ± 0.28. *p* < 0.05). However, the values of PT (days 14, 30, and 90), TT (days 14, 30, 60, and 90), and APTT (days 14, 30, 60, and 90) in the sham group were higher than those in the IPC group (PT: sec, sham group, d14: 9.71 ± 1.40, d30: 9.46 ± 1.28, d90: 10.07 ± 1.48, IPC group, d14: 10.36 ± 1.58, d30: 9.76 ± 1.65, d90: 10.58 ± 1.88. TT: sec, sham group, d14: 16.05 ± 4.11, d30: 15.57 ± 3.99, d60: 16.34 ± 2.77, d90: 17.49 ± 4.23, IPC group, d14: 18.15 ± 3.78, d30: 16.59 ± 4.75, d60: 17.67 ± 3.52, d90: 18.17 ± 3.56. APTT: sec, sham group, d14: 23.41 ± 5.28, d30: 23.92 ± 3.76, d60: 24.97 ± 4.71, d90: 24.49 ± 5.25, IPC group, d14: 24.78 ± 4.79, d30: 25.03 ± 4.52, d60: 26.61 ± 5.04, d90: 26.91 ± 5.39. *p* < 0.05).

**TABLE 2:** Serum coagulation function levels.

		IPC	Sham	T value	*P*-value
PT	Before PICC	10.31 ± 1.36	10.26 ± 1.31	0.474	0.636
	14 days after PICC	10.36 ± 1.58	9.71 ± 1.40	5.292	<0.001*
	30 days after PICC	9.76 ± 1.65	9.46 ± 1.28	2.498	0.013*
	60 days after PICC	10.19 ± 1.92	10.00 ± 1.57	1.334	0.183
	90 days after PICC	10.58 ± 1.88	10.07 ± 1.48	3.660	<0.001*
TT	Before PICC	17.93 ± 3.12	18.30 ± 3.11	1.438	0.151
	14 days after PICC	18.15 ± 3.78	16.05 ± 4.11	6.505	<0.001*
	30 days after PICC	16.59 ± 4.75	15.57 ± 3.99	2.825	0.005*
	60 days after PICC	17.67 ± 3.52	16.34 ± 2.77	5.149	<0.001*
	90 days after PICC	18.17 ± 3.56	17.49 ± 4.23	2.138	0.033*
APTT	Before PICC	27.26 ± 4.63	26.62 ± 4.56	1.704	0.089
	14 days after PICC	24.78 ± 4.79	23.41 ± 5.28	3.336	0.001*
	30 days after PICC	25.03 ± 4.52	23.92 ± 3.76	3.249	0.001*
	60 days after PICC	26.61 ± 5.04	24.97 ± 4.71	4.099	<0.001*
	90 days after PICC	26.91 ± 5.39	24.49 ± 5.25	5.560	<0.001*
FDP	Before PICC	1.80 ± 1.08	1.91 ± 1.56	1.001	0.317
	14 days after PICC	2.53 ± 1.57	3.07 ± 1.91	3.770	<0.001*
	30 days after PICC	2.54 ± 1.69	3.11 ± 1.74	4.044	<0.001*
	60 days after PICC	2.01 ± 1.76	2.31 ± 1.51	2.236	0.026*
	90 days after PICC	1.96 ± 1.58	2.20 ± 1.62	1.822	0.069*
D-Dimer	Before PICC	0.28 ± 0.25	0.29 ± 0.28	0.438	0.661
	14 days after PICC	0.31 ± 0.39	0.33 ± 0.31	0.707	0.480
	30 days after PICC	0.36 ± 0.45	0.39 ± 0.45	0.689	0.491
	60 days after PICC	0.26 ± 0.28	0.32 ± 0.26	2.478	0.013*
	90 days after PICC	0.28 ± 0.33	0.29 ± 0.33	0.344	0.731

*p < 0.05 compared with the sham group.

### The observation of PICC-related complications

**FIGURE 2:**
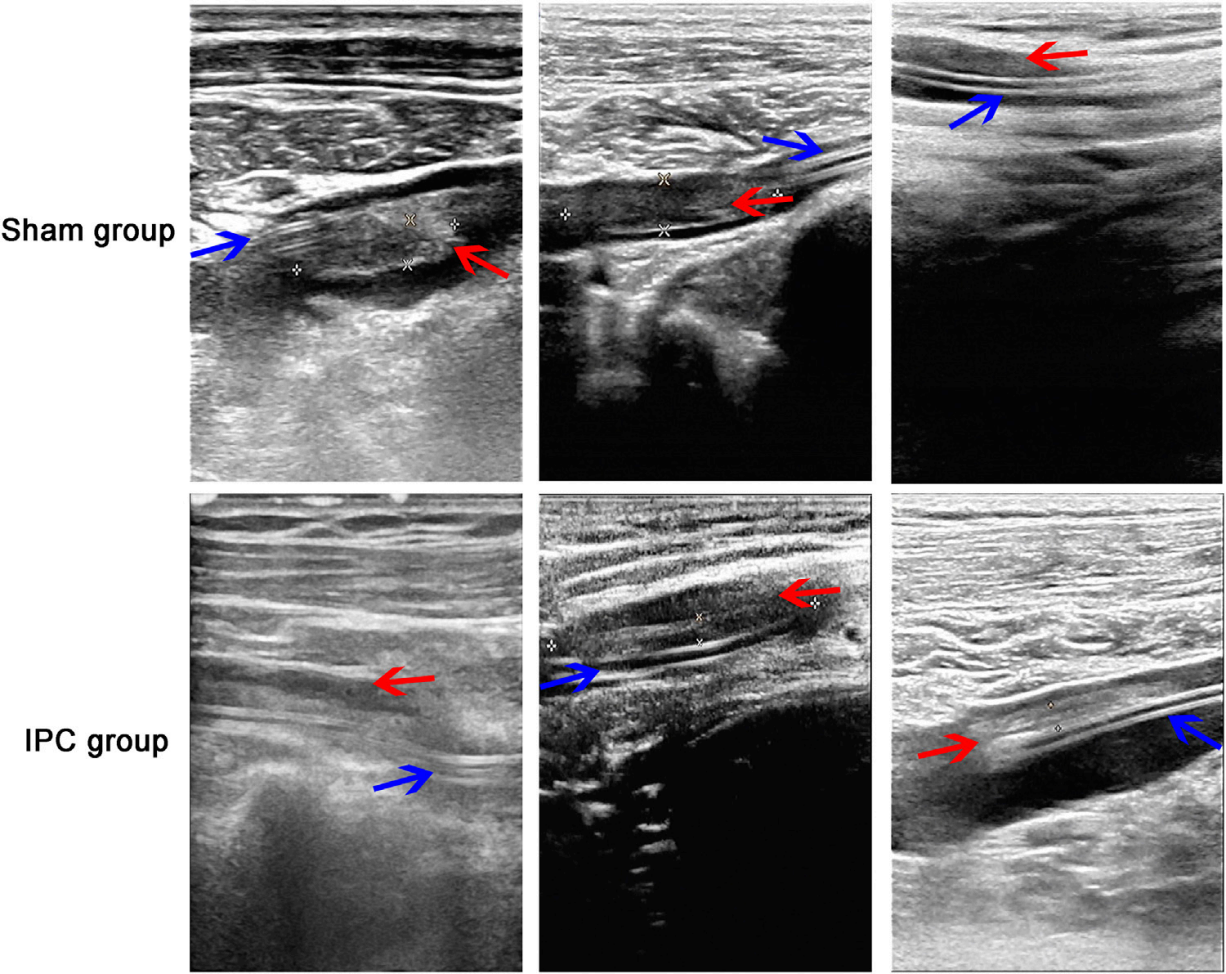
The observation of thrombosis. The absence of PICC-related thrombosis in the sham group was more than that in the IPC group, Substantial hypoechoic and irregular blood flow signals were observed in the vascular lumen of the sham group, and the incidence rate of thrombosis in the IPC group was less than that in the sham group (*p < 0.05 compared with the sham group. Red arrow: thrombosis; blue arrow: catheter).

PICC-related complications were observed and recorded, and the incidence rate of the thrombosis was assessed by color Doppler ultrasound at days 14, 30, 60, and 90 after PICC. Moreover, the thrombus was observed on grayscale imaging in all 600 patients. The results revealed that 56/300 patients in the sham group had presence of PICC-related thrombosis, with only 23/300 in the IPC group (Figure 2). No significant difference was found in other complications between the two groups (Table 3).

**TABLE 3:** The observation of related complications.

Type of complication		IPC	Sham	X^2^ value	*P*-value
Local inflammation	n (%)	21 (7)	22 (7.3)	0.025	0.87
Bloodstream infection	n (%)	4 (1.3)	5 (1.7)	0.11	0.74
Occlusion	n (%)	3 (1)	3 (1)	0	1
Dislocation	n (%)	2 (0.7)	1 (0.3)	0.34	0.56
PICC-related thrombosis	n (%)	23 (7.7)	56 (18.7)	15.88	<0.05*
Local bleeding	n (%)	0 (0)	1 (0.3)	1	0.32
Pulmonary artery embolism	n (%)	0 (0)	0 (0)	0	1
Local allergic reaction	n (%)	1 (0.3)	1 (0.3)	0	1

*p < 0.05 compared with the sham group.

### Length of hospital stay

To evaluate the effects of PICC treatment, the length of hospital stay was compared between the two groups. All the 600 individuals were admitted to the hospital 3 times after PICC for chemotherapy. The hospital stays are shown in Table 4, with the hospital stays in the IPC group being shorter than in the sham group [Table 4, hospital stays (day), sham group: 6.77 ± 3.15. IPC group: 6.16 ± 3.03. *p* < 0.05].

**TABLE 4:** Hospital stays, hospitalization expenses and patient satisfaction.

	IPC	Sham	T value	*P*-value
Hospital stays(day)	6.16 ± 3.03	6.77 ± 3.15	2.15	<0.05*
Hospitalization expenses(Yuan)	17129.596 ± 2351.66	18032.66 ± 1911.64	6.83	<0.05*
Patient satisfaction(%)	95.77 ± 3.66	91.9 ± 5.24	6.66	<0.05*

*p < 0.05 compared with the sham group.

### Hospitalization expenses and patient satisfaction

The cost of hospitalization was also reduced in the IPC group when compared with the sham group, which also improved the satisfaction of patients [Table 4, hospitalization expenses (Yuan), shan group: 18032.66 ± 1911.64, IPC group: 17129.596 ± 2351.66. Patient satisfaction (%), sham group: 91.9 ± 5.24, IPC group: 95.77 ± 3.66. *p* < 0.05].

## Discussion

This study provides useful insights into the effect of limb ischemic preconditioning on the incidence of PICC-related thrombosis in patients with peripherally inserted central catheters. Limb ischemic preconditioning may attenuate the severity of vein thrombosis in patients with peripherally inserted central catheters. The coagulation function levels (PT, TT, APTT, FDP, and D-Dimer) in patients of the sham and IPC groups were first compared, revealing that the values of PT, TT, APTT, FDP, and D-Dimer in patients of the IPC group were normal, but abnormal changes were detected in the coagulation function indicators in the sham group. Then the incidence of PICC-related complications was assessed, displaying a decreased incidence in the IPC group compared with the sham group. The incidence of PICC-related thrombosis in the IPC group was reduced when compared with the sham group, but no significant difference in other complications was observed between the two groups. In addition, the hospital stays were longer in patients of the sham group. Finally, the cost of hospitalization was also lower in the IPC group compared with the sham group, which also improved the satisfaction of patients.

Several approaches to venous access are used in our clinic, including centrally inserted venous catheters as well as peripherally inserted central catheters. When compared with the centrally inserted venous catheters, PICC showed some advantages in the administration of antibiotics, total parenteral nutrition, chemotherapy, fluid replacement, and drug administration. PICC is convenient, easy to operate, safe to insert, and carries a low risk of vascular injury or blood infection. Nevertheless, PICC can also lead to complications, such as infection, phlebitis, and thrombosis. Among these complications, PICC-related thrombosis is relatively common, which may further lead to a potentially serious complication, such as pulmonary embolism. Therefore, proper evaluation and management of PICC are critical for patient health and prognosis, and finding ways to reduce the incidence of catheter-related thrombosis is the main emphasis of our research [13–17].

Ischemic preconditioning was reported to play a protective role in reducing platelet activation, possibly exerting favorable effects on the occurrence of thromboembolic events. Ischemic preconditioning is a process by which the brief periods of ischemia in a tissue protect another organ or tissue. This procedure is widely used and has been verified in our previous study [18–20]. Platelet-derived extracellular vesicles (PEVs), being small particles, extracellular vesicles are associated with various diseases including inflammation, vascular disorders, and tumors. PEVs function similarly to platelets and positively affect hemostasis, thrombus formation, and pro-inflammatory processes through different mechanisms. Additionally, research has discovered that pre-treating limb ischemia can decrease the production of platelet-derived extracellular vesicles which consequently exerts a favorable impact on reducing vascular inflammation and thrombus formation [21]. The current study focused on the process of IPC and thrombus recanalization in patients after PICC. Firstly, PICC was successfully established, and the process was confirmed by an x-ray, which showed the position of the catheter tip. All the patients underwent the PICC procedure successfully.

Coagulation function tests (PT, TT, APTT, FDP, D-dimer, etc.) can effectively evaluate the body’s coagulation status. Research has shown that a hypercoagulable state slows down blood flow and promotes the formation of venous thrombosis. PT, TT, APTT, FDP, D-dimer and other indicators reflect the hypercoagulable state of the body through different mechanisms. For example, TT can indicate the presence of pathological anticoagulant substances in circulating blood; PT is mainly used to reflect the exogenous coagulation pathway; APTT is used to reflect the endogenous coagulation pathway; TT and FDP are primarily used to reflect common coagulation pathways’ status. D-dimer is a specific degradation product produced by fibrin monomers during fibrinolysis. An elevated level of D-dimer reflects secondary fibrinolysis activation. FDP and D-dimer are specific indicators for evaluating high-coagulation states and excessive fibrinolysis *in vivo* as well as assessing pre-thrombotic hypercoagulability. Therefore, testing coagulation-related indicators at different time points allows assessment of changes in overall coagulation status to evaluate relative risk of thrombus formation [22–24].

Subsequently, the coagulation function was evaluated, revealing that PT, TT, APTT, FDP, and D-Dimer in patients of the IPC group were more normal than that in patients of the sham group. All of the values were within the normal reference range, but the values of FDP (days 14, 30, 60, and 90) and D-Dimer (day 60) in the sham group were significantly higher than those in the IPC group (*p* < 0.05). However, the values of PT (days 14, 30, and 90), TT (days 14, 30, 60, and 90), and APTT (days 14, 30, 60, and 90) in the sham group were higher than in the IPC group (*p* > 0.05). Increased PT and APPT values were detected in the sham group after the thrombus was confirmed, which was due to the antithrombotic drugs (such as low molecular weight heparin, warfarin, and so on). Some reports confirmed that IPC could reduce platelet-fibrinogen binding, platelet-neutrophil aggregates, and platelet P-selectin expression, which results in the suppression of platelet activation [12]. All of the above was in accordance with our results.

Secondly, PICC-related complications were observed and recorded. A lower incidence of PICC-related thrombosis was observed in the IPC group compared with the sham group. 56/300 patients in the sham group had presence of PICC-related thrombosis, with only 23/300 in the IPC group. No significant difference in other complications was observed between the two groups, including local inflammation, bloodstream infection, occlusion, dislocation, local bleeding, pulmonary artery embolism, and local allergic reaction. Our results were consistent with the previous study by Ye Sun et al. [25], which suggested that localized lower extremity IPC could reduce deep vein thrombosis formation in a rat experimental thrombosis model. The anti-thrombotic effects of IPC could be attributed to the inhibition of platelet activation [9].

Furthermore, the length of hospital stay was compared between the two groups, showing longer hospital stays in the sham group. The cost of hospitalization was also lower in the IPC group than in the sham group, which also improved the satisfaction of patients.

The mechanism underlying the decreased thrombosis in IPC remains elusive but may involve the release of the antithrombotic substances from the vessel wall, as well as the suppressed effects of IPC on platelets, coagulation, and fibrinolysis. NO (nitric oxide) is a key damaging factor in ischemia/hypoxia, exhibiting dual effects on the body’s protection. During ischemia/hypoxia, it can both improve blood supply to the injured area by dilating local blood vessels and inhibit platelet aggregation and leukocyte adhesion to protect tissues, as well as damage the body by causing DNA injury and generating more toxic oxygen free radicals. NOS is the crucial enzyme for NO synthesis, with three subtypes: neuronal nitric oxide synthase (nNOS), endothelial nitric oxide synthase (eNOS), and inducible nitric oxide synthase (iNOS). When the body experiences ischemia, eNOS activity increases, leading to increased NO production. This causes vasodilation in the ischemic area, improving blood supply and providing protection for ischemic tissues [26, 27]. The protective effects of IPC have yet to be described in animals, and this study further confirmed the action of IPC on thrombosis downregulation in humans. However, the specific experimental mechanism of this protective function requires further animal studies [9, 12, 28–31].

In conclusion, the present study demonstrated that limb ischemic preconditioning may prevent vein thrombosis in patients with peripherally inserted central catheters, which may improve their quality of life and reduce the length of hospitalization. However, the specific mechanism requires further research, and the application of IPC on patients with a high risk of deep venous thrombosis also needs to be analyzed.

## Data Availability

The raw data supporting the conclusions of this article will be made available by the authors, without undue reservation.

## Abbreviations

IPC, Limb ischemic preconditioning; PICC, peripherally inserted central catheter; SOD, superoxide dismutase; NO, nitric oxide.
